# The competitive advantage of institutional reward

**DOI:** 10.1098/rspb.2019.0001

**Published:** 2019-03-27

**Authors:** Yali Dong, Tatsuya Sasaki, Boyu Zhang

**Affiliations:** 1 School of Systems Science, Beijing Normal University, Beijing 100875, People’s Republic of China; 2 F-power Inc., Roppongi 1-8-7-2F, Minato, Tokyo 106-0032, Japan; 3 Laboratory of Mathematics and Complex Systems, Ministry of Education, School of Mathematical Sciences, Beijing Normal University, Beijing 100875, People’s Republic of China

**Keywords:** Cooperation, evolutionary game, reward, punishment, efficiency, decision errors

## Abstract

Sustaining cooperation among unrelated individuals is a fundamental challenge in biology and the social sciences. In human society, this problem can be solved by establishing incentive institutions that reward cooperators and punish free-riders. Most of the previous studies have focused on which incentives promote cooperation best. However, a higher cooperation level does not always imply higher group fitness, and only incentives that lead to higher fitness can survive in social evolution. In this paper, we compare the efficiencies of three types of institutional incentives, namely, reward, punishment, and a mixture of reward and punishment, by analysing the group fitness at the stable equilibria of evolutionary dynamics. We find that the optimal institutional incentive is sensitive to decision errors. When there is no error, a mixture of reward and punishment can lead to high levels of cooperation and fitness. However, for intermediate and large errors, reward performs best, and one should avoid punishment. The failure of punishment is caused by two reasons. First, punishment cannot maintain a high cooperation level. Second, punishing defectors almost always reduces the group fitness. Our findings highlight the role of reward in human cooperation. In an uncertain world, the institutional reward is not only effective but also efficient.

## 1. Introduction

‘How did cooperative behaviour evolve’ is a fundamental question in biology and the social sciences [1]. Although kin selection and direct reciprocity provide satisfactory explanations of cooperation in small groups, the sustaining of collective efforts in sizable groups of unrelated individuals remains a problem [2,3]. Examples such as the public goods game (PGG) show that self-interested players should prefer a free-rider strategy, whereas it is to everyone’s advantage for all players to contribute [4]. In real-life situations that include this sort of dilemma between individual rationality and collective advantage, incentives are often used to promote cooperation, where contributors may be rewarded and free-riders may be punished. In fact, positive and negative incentives exist not only in human societies but also in animal behaviour [5]. Understanding the consequences of such ‘carrots and sticks’ in boosting cooperation is a core topic in the evolutionary biology and behavioural sciences (see [6–8] for three recent review papers). Most previous studies have focused on which incentives promote cooperation best. However, in the evolutionary process, groups with higher fitness rather than a higher cooperation level can survive. We thus argue that a better measurement for the success of an incentive is the group average fitness at the evolutionary stable state. Currently, it is unclear which type of incentive is more efficient in preserving group income. Specifically, when individuals are boundedly rational and may make mistakes in decision-making, is it better to use reward or punishment? In this paper, we apply evolutionary game theory to address these questions.

In past decades, several types of incentives have been proposed to promote cooperation in social dilemma games. Most of these investigations addressed so-called peer (or decentralized) incentives, and only a minority considered so-called institutional (or centralized) incentives. In the peer incentive scenario, players are allowed to reward and/or punish others at a cost to themselves (for experimental studies, see, e.g. [9–25]; for theoretical studies, see, e.g. [26–35]). Laboratory experiments have indicated that peer incentives can curb free-riding in human populations. However, this paradigm suffers from several drawbacks. First, the use of incentives is costly, which then raises an issue of second-order free-riding. In fact, the incentive system itself is a common good that can be exploited, and reward and punishment schemes are individually disadvantageous [18]. Second, some players abuse sanctioning opportunities by engaging in antisocial punishment, which harms cooperators [14,15,18,20,22]. In the institutional incentive scenario, it is not individuals who reward or punish. Rather, an institution rewards and punishes individuals based on their contributions (for experimental studies, see, e.g. [36–42]; for theoretical studies, see, e.g. [43–51]). Institutional incentives can overcome the problem of second-order free-riding and avoid antisocial punishment. However, this approach is more wasteful than peer incentives because subjects have to pay a fee to maintain the institution even if no one is being rewarded or punished (which can be viewed as paying for the upkeep of a police force).

The use of institutional incentives is a common feature in many parts of human society such as government institutions and businesses. Theoretical studies based on evolutionary game theory have revealed that the effect of institutional incentives on cooperation can be understood in terms of the incentive size [44,46,47,49]. If the incentive is very small, then both reward and punishment have no effect on promoting cooperation, and selfish players maintain a free-riding strategy. If the incentive is sufficiently large, then both reward and punishment compel all players to cooperate. If the incentive is intermediate, then full contribution can become evolutionarily stable under punishment, and reward can cause only the stable coexistence of free-riders and cooperators. Although punishment promotes cooperation better than a reward for intermediate incentives, this approach appears more socially expensive. In fact, a rewarding institution can increase the payoff of individuals, whereas a punishment institution cannot. Furthermore, when there are free-riders in the group, the decrease in payoffs through punishment may exceed the gains increased from cooperation, which results in an overall reduction of the social welfare [39,42,46].

In this paper, we consider three types of institutional incentives, namely, reward, punishment, and a mixture of reward and punishment, and investigate which type of incentive is more efficient in preserving group income. Following [39,42,43,46,47], players of a PGG with institutional incentives have to pay a fee to the institution before the joint enterprise takes place, and the institution rewards cooperators and punishes defectors. The incentive institution is established in advance and thus entails running costs even in the case that no one deserves reward or punishment [39,42,43,46]. To better simulate reality, we assume that individuals are boundedly rational, that is, they preferentially imitate the successful strategies and may make mistakes in decision-making [42,43,52,53]. This learning process can be described by the replicator-mutator equation [54]. We compare the efficiencies of the three types of incentives by analysing the population average fitness at the stable equilibria of the replicator-mutator equation. The main result is that the efficiencies are sensitive to decision-making errors. Without decision errors, a mixture of reward and punishment can maintain a high cooperation level and high group average fitness. However, for intermediate or large decision errors, the use of punishment almost always reduces the group welfare, and reward becomes the most efficient incentive.

## 2. Material and methods

Consider an infinitely large and well-mixed population; from time to time, a sample of size *n* is randomly chosen to form a PGG [46,47]. In the PGG, each player can decide whether to contribute a fixed amount *c* > 0 knowing that this amount will be multiplied by *r* > 1 and divided equally among all *n* players in the group. If *n*_*C*_ is the number of those players who contribute (i.e. cooperators) and *n*_*D*_ the number of those who do not (i.e. defectors), then the payoffs of a cooperator and a defector are *rc*(*n*_*C*_)/*n* − *c* and *rc*(*n*_*C*_)/*n*, respectively. In a PGG with institutional incentive, each player has to pay *C*_I_ for the institution, and the total amount of incentive is *C*_I_*n*. In the case of institutional reward (IR), the incentive is shared among the *n*_*C*_ cooperators. Thus, each cooperator obtains a reward of (*C*_I_*n*)/(*n*_*C*_). In the case of institutional punishment (IP), each defector analogously receives a punishment of (*C*_I_*n*)/(*n*_*D*_). Finally, we consider an incentive institution that can provide both reward and punishment (IRP). Assume that *α* of the total incentive is used for reward and that the remaining 1 − *α* is used for punishment. Thus, the payoff of each cooperator is increased by *α*(*C*_I_*n*)/(*n*_*C*_), and the payoff of each defector is decreased by (1 − *α*)(*C*_I_*n*)/(*n*_*D*_). In particular, *α* = 0 and *α* = 1 correspond to the cases of IP and IR, respectively. Therefore, IRP with *α* provides a more general framework for studying institutional incentives.

We apply evolutionary game theory to study the evolution of cooperation in PGG with institutional incentive [4,54,55]. Let *x* be the frequency of cooperators in the population and *P*_C_ and *P*_D_ be the expected payoffs of a cooperator and a defector in a randomly formed PGG, respectively (see electronic supplementary material, §1.1 for payoff calculation). We then define the expected fitness of a cooperator and a defector by *f*_C_ and *f*_D_, respectively, where *f*_C_ = *ωP*_C_ + 1 − *ω* and *f*_D_ = *ωP*_D_ + 1 − *ω*. The parameter *ω* ∈ [0, 1] is interpreted as a selection intensity [54,55]. If *ω* = 0, then the payoff of the PGG does not contribute to fitness. If *ω* = 1, then the fitness is entirely determined by the payoff. Specifically, a positive *ω* does not change the direction of evolution but only affects the speed.

We consider that individuals update their strategies based on the preferential imitation of more successful strategies, where a strategy with a higher fitness is expected to spread. This process can be described by the following replicator equation:

2.1 $$\frac{dx}{dt}=x(1-x)(f_{C}-f_{D}).$$

We now allow decision errors and assume that an individual adopts a random strategy with probability *μ*. Equation (2.1) can then be modified as

2.2 $$\frac{dx}{dt}=x(1-x)(f_{C}-f_{D})+\frac{\mu }{2}(1-x)-\frac{\mu }{2}x.$$

Equation (2.2) is called the replicator-mutator equation [54]. Biologically, this equation describes a simultaneous action of selection and mutation. Specifically, the two effects are independent, where the first term *x*(1 − *x*)(*f*_*C*_ − *f*_*D*_) is the selection term and the second term *μ*/2(1 − *x*) − *μ*/2*x* is the mutation term [54].

## 3. Results

### (a) Without decision errors

If players do not make a mistake, both the defective state *x* = 0 and the cooperative state *x* = 1 are equilibria for equation (2.1). In particular, the defective equilibrium is globally stable when there is no incentive, which corresponds to the state that all individuals in the population are defectors.

**Figure 1.**
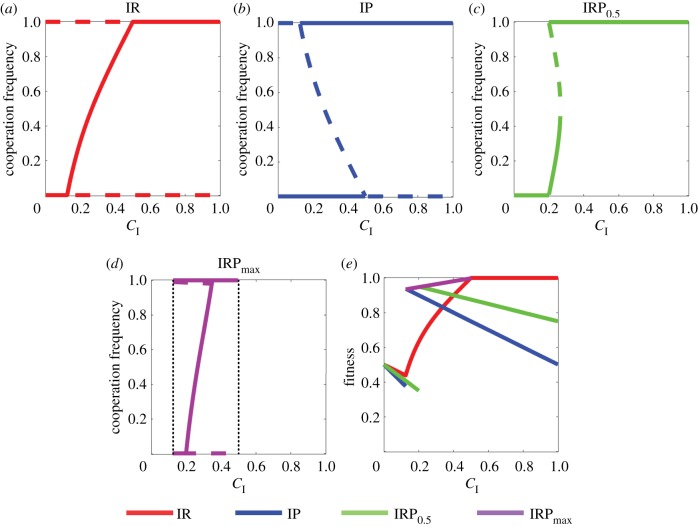
PGG without decision errors. Parameters are taken as *n* = 4, *c* = 1, *r* = 2, *ω* = 0.5, and *μ* = 0. (*a*)–(*d*) Equilibria of equation (2.1) with different types of incentives, where stable equilibria are denoted by solid curves and unstable equilibria are denoted by dashed curves. (*a*) IR has a globally stable equilibrium. (*b*) For IP, the cooperative and the defective equilibria are bistable. (*c*) IRP may have two interior equilibria in addition to the two boundary equilibria. (*d*) IRP_max_ is defined for 0.125 < *C*_I_ < 0.5 (see the dotted lines), and the cooperative equilibrium is stable in this interval. (*e*) Group average fitness at the stable (cooperative) equilibria for different types of incentives. Overall, the reward can maximize the group average fitness for small and large incentives, and a mixture of reward and punishment performs best for intermediate incentives. (Online version in colour.)

We first analyse the stability of equation (2.1) for different types of incentives (see electronic supplementary material, §1.2 for equilibria calculation and stability analysis). The cases of IR and IP were previously investigated in [47]. For both cases, if *C*_I_ is greater than an upper bound $C{{\;}_{I}}^{+}=c-(rc)/n$, then the existence of an interior equilibrium is impossible, and the cooperative equilibrium *x* = 1 is globally stable. By contrast, if *C*_I_ is smaller than a lower bound $C_{I}^{\;\;\;-}=c/n-(rc)/(n^{2})$, then the defective equilibrium *x* = 0 is globally stable. Therefore, we focus mainly on $C{{\;}_{I}}^{-}<C{\;}_{I}<C{{\;}_{I}}^{+}$ in later discussions, i.e. the incentive size is intermediate in the sense that it has some effects on promoting cooperation but is not sufficient to guarantee the global stability of the cooperative equilibrium. For IR with intermediate incentive, both the cooperative and the defective equilibria are unstable, and equation (2.1) has a globally stable interior equilibrium x*_R_ (see figure 1*a*). For IP with intermediate incentive, the cooperative and the defective equilibria are bistable, and equation (2.1) has an unstable interior equilibrium x*_P_ (see figure 1*b*). Finally, for IRP with intermediate incentive, equation (2.1) may have (at most) two interior equilibria in addition to the two boundary equilibria (figure 1*c*). Furthermore, the cooperative equilibrium is stable if and only if *C*_I_ > ((*n* − *r*)*c*)/(*n*(*α* + (1 − *α*)*n*)).

We next look at the efficiencies of the three types of incentives by comparing their average fitness at the stable (cooperative) equilibria. First, although IP can maintain full contribution, whether this strategy is more efficient than IR depends on the incentive size *C*_I_. In fact, in IP, the average fitness is maximized when *C*_I_ barely exceeds the lower bound *C*_I_^−^, while in IR, the maximum fitness is achieved when *C*_I_ reaches the upper bound *C*_I_^+^ [46]. Second, IRP is more efficient than IP whenever the cooperative equilibrium is stable. This result implies that a mixture of reward and punishment works better than punishment only. Finally, whether IRP has a higher fitness than IR depends on both *C*_I_ and *α* (see figure 1*d*). For intermediate incentive, the fitness of IRP is maximized when *α*_max_ = (*n* − (*n* − *r*)*c*/*nC*_I_)/(*n* − 1), i.e. the maximum *α* such that the cooperative equilibrium is stable. With this *α*_max_, IRP must be more efficient than IR (see figure 1*e*).

We now show the optimal incentive that maximizes the group average fitness at the stable (cooperative) equilibrium. If $C{\;}_{I}\leq C_{I}^{\;\;-}$, then the defective state is the only stable equilibrium. In this case, the optimal incentive is IR because the use of punishment decreases the social welfare. On the other hand, if $C_{I}\geq C_{I}^{\;\;+}$, then the cooperative state is the only stable equilibrium. The optimal incentive in this case is also IR because the punishment institution is costly even if no one is punished. Finally, if $C_{I}^{\;\;-}<C_{I}<C_{I}^{\;\;+}$, then the optimal incentive is IRP with *α*_max_ (denoted by IRP_max_). Overall, reward alone is the most efficient incentive for both small and large *C*_I_, and a mixture of reward and punishment is the most efficient incentive for intermediate *C*_I_ (see figure 1*e*).

### (b) With decision errors

**Figure 2.**
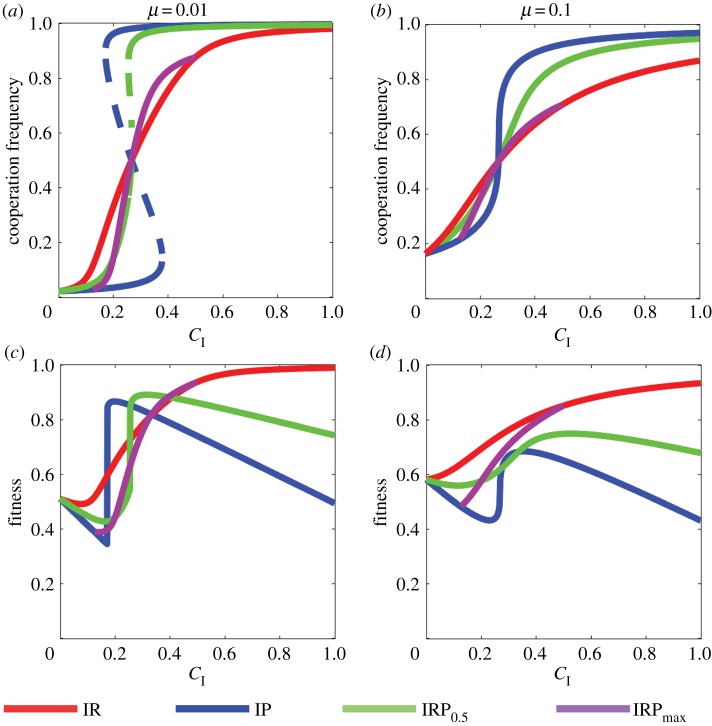
PGG with decision errors. Parameters are taken as *n* = 4, *c* = 1, *r* = 2, *ω* = 0.5. (*a*),(*b*) Equilibria of equation (2.2) with different types of incentives and decision errors, where stable equilibria are denoted by solid curves and unstable equilibria are denoted by dashed curves. IRP_max_ is defined for 0.125 < *C*_I_ < 0.5. (*a*) For small errors, IR and IRP_max_ have a unique stable equilibrium, and IP and IRP_0.5_ may have three equilibria. (*b*) For intermediate errors, all the incentives have a unique stable equilibrium. (*c*),(*d*) Group average fitness at the stable (cooperative) equilibria for different types of incentives and decision errors. The optimal incentive is sensitive to decision errors. (*c*) For small errors, the incentives that lead to the highest group average fitness are, from *C*_I_ = 0 to 1, IR, IP, IRP_0.5_, IRP_max_, and IR. (*d*) For intermediate errors, IR performs best for all *C*_I_. (Online version in colour.)

Decision errors can dramatically change the evolutionary process. Foremost, when subjects can make mistakes, the cooperative state and the defective state are no longer in equilibria. As a result, cooperators and defectors stably coexist, and no incentive can maintain full cooperation. Second, equation (2.2) has a unique and globally stable interior equilibrium for larger *μ* and smaller *ω*. In particular, a crucial *C*_I_* exists such that IR leads to the highest cooperation level for $C_{I}<C_{I}^{\;\;*}$ and that IP is the most effective incentive in promoting cooperation for $C_{I}>C_{I}^{\;\;*}$ (see figure 2*b*; electronic supplementary material, § 1.3).

We further study the properties of the equilibria of equation (2.2) through numerical simulations (see electronic supplementary material, § 3.1 for simulation codes). Figures 2*a*,*b* show equilibria of equation (2.2) for different types of incentives under small and intermediate decision errors. On the one hand, both IR and IP are robust to small *μ*, i.e. IR has a globally stable equilibrium for all *C*_I_ and IP has two stable equilibria and one unstable equilibrium for intermediate *C*_I_. However, IRP is sensitive to *μ*. As shown in figure 2*b*, IRP_max_ is more like IR with its unique equilibrium, and IRP with *α* = 0.5 (denoted by IRP_0.5_) is more like IP, which can have three equilibria. On the other hand, all the incentives have a unique globally stable equilibrium for intermediate *μ*. Specifically, *C*_I_ plays a predominant role in determining the equilibrium value, where the cooperation rate is continuously increasing in *C*_I_. Finally, IRP is never superior in promoting cooperation; rather, it is dominated by IR for smaller *C*_I_ and by IP for larger *C*_I_.

**Figure 3.**
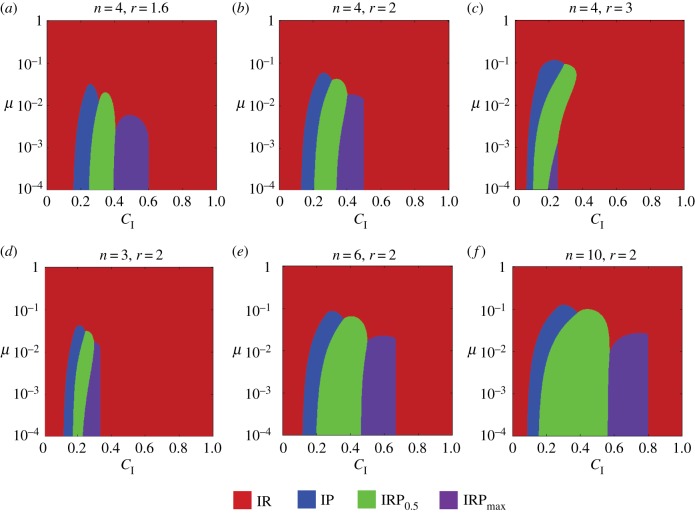
Optimal incentives for different *C*_I_, *μ*, *n*, and *r*. *c* = 1 and *ω* = 0.5 in all six subfigures. IR, IP, IRP_0.5_, and IRP_max_ lead to the highest group average fitness in the red, blue, green, and purple regions, respectively. For small errors, the incentives that lead to the highest group average fitness are, from *C*_I_ = 0 to 1, IR, IP, IRP_0.5_, IRP_max_, and IR. For intermediate and large errors, IR is the optimal incentive for all *C*_I_, *n* and *r*.

We next compare the group average fitness for different types of incentives. For small decision errors *μ* = 0.01, the optimal incentive is IR for small and large *C*_I_ and is IP or IRP for intermediate *C*_I_ (see figure 2*c*). Notably, as *μ* increases to 0.1, IR becomes the most efficient incentive for all *C*_I_ (see figure 2*d*). Figure 3 systematically illustrates the effect of decision errors on incentive efficiencies, indicating that the above observations are robust against a variety of *n* and *r*. Overall, for small decision errors, the optimal incentives are, from *C*_I_ = 0 to 1, IR, IP, IRP_0.5_, IRP_max_, and IR. If we exclude IRP_max_, then this order is consistent with the case of *μ* = 0. This result reveals that the efficiencies of IR, IP, and IRP with constant *α* are stable against small mutations. By contrast, IRP_max_ can no longer maintain high fitness even for small decision errors. Finally, for intermediate and larger decision errors, IR leads to the highest group average fitness for all *C*_I_, *n* and *r*.

### (c) Robustness analysis

In electronic supplementary material, §2, we evaluate the robustness of the results with respect to the following model variants. (i) We consider an ‘others only’ variant of the PGG (also called a ‘mutual aid’ game) in which a cooperator does not benefit from its own contribution [4,43,46,56–58]. (ii) The incentive institution can be modelled in different ways. On the one hand, one may assume that the total amount of reward is multiplied by a factor *r*_1_ before it is distributed among cooperators, and the total amount of punishment is multiplied by a factor *r*_2_ before it is distributed among defectors [33,35]. On the other hand, the incentive could be probabilistic where only one cooperator (or defector) is exemplarily rewarded (punished) [36,40,41,44]. (iii) The institution makes a mistake in incentive distribution [47]. For instance, the institution may punish a cooperator or reward a defector due to a lack of information or observation errors. We find that none of the variants (i)–(iii) qualitatively affect our results regarding the equilibria of the evolutionary dynamics and the efficiencies of the different types of incentives. For intermediate or large decision errors, the optimal incentive is IR for all *C*_I_.

## 4. Discussion

Reward and punishment are often used to promote cooperation in social dilemmas. Most of the previous studies have focused on which type of incentive best promotes cooperation (see, e.g. [6–8,33]). However, a high cooperation level does not always lead to high fitness. For instance, punishment can effectively promote cooperation, but experiments rarely find a significant increase in net payoff in that context [11,18,23]. In this paper, we argue that a better measurement for the success of an incentive is the group average fitness at the evolutionary stable state. The reason is twofold: on the one hand, the group average fitness plays a predominant role in the evolutionary process. In fact, only groups with higher fitness can win the competition for survival, and groups with lower fitness tend to go extinct. On the other hand, if players update strategies based on payoff-driven decision rules such as ‘best-response’ or ‘imitate-the-better’ (these two decisions rules have been widely used in the previous studies, e.g. [4,43–49]), then the players should also prefer the incentive institution that can lead to a higher income rather than a higher cooperation level.

In this paper, we compare the efficiencies of three types of institutional incentive, namely, reward, punishment, and a mixture of reward and punishment. We emphasize that players are boundedly rational and may make mistakes in decision-making. Recent studies in neuroscience revealed that errors are inevitable in human decision-making, even reward and punishment cannot eliminate unintended errors [59]. Therefore, it is important to investigate how decision errors affect the efficiencies of different types of incentives. By analysing the group average fitness at the stable equilibria of the replicator-mutator equation, we find that the optimal incentive is sensitive to decision errors. Without decision errors, the reward can maximize the group average fitness for small and large incentives, and a mixture of reward and punishment performs best for intermediate incentives. However, if players make mistakes in decision-making, then the use of punishment almost always reduces the group welfare, and the reward is revealed as the most efficient incentive for intermediate and large decision errors. Numerical simulations show that this result is robust in the standard PGG and alternative model variants.

The failure of IP and IRP is caused by two reasons. On the one hand, these strategies can no longer maintain a high cooperation level when error exists. As shown in figures 1 and 2, the stable equilibria in IP and IRP with *μ* = 0.1 are different from that of *μ* = 0 and *μ* = 0.01. For smaller *C*_I_, the cooperation levels in IP and IRP are always lower than that of IR. On the other hand, punishing defectors significantly reduces the group fitness. This problem is not severe when players do not make mistakes. Because in this case, full cooperation can be stable in IP and IRP, and no one is punished. However, for intermediate or large decision errors, the proportion of defectors is not rare, and the loss of punishment is typically inevitable.

We note that the effects of institutional incentives and peer incentives in promoting group fitness are substantially different. On the one hand, intermediate errors can increase cooperation and improve the group average fitness in the PGG with peer punishment [52,60]. However, errors are harmful to institutional punishment. On the other hand, (budget-balanced) peer reward can never lead to a high cooperation level [4,26,30], but the institutional reward can promote cooperation regardless of the presence of errors. This result implies that institutional rewards have advantages over other types of incentives, as they can promote cooperation without hurting the group welfare and are robust against decision errors.

In our model, every player has to pay for the incentive institution before contributing to the PGG. In fact, this payment can be seen as an entry fee for the PGG. If the payment is voluntary, then the issue of second-order free-riding is raised because the incentive institution itself is a common good that can be exploited. Previous studies show that IP with voluntary payment is functional if punishment is also imposed on second-order free-riders (i.e. subjects who cooperate but do not pay for the institution) [39,42,43]. By contrast, if IR is budget-balanced, then rational players will not pay for the institution [35]. Since the entry fee in our model is compulsory, a subsequent question is whether real players will choose such an incentive institution. A recent experimental study indicated that approximately half of the subjects would like to pay 20% of their wealth for a reward mechanism before playing the PGG [61]. This finding suggests that the incentive institution we proposed could be sustainable.

In addition, the incentive institution considered in this paper is not adaptive, i.e. *α* does not change over the cooperation level. Notably, a ‘first carrot, then stick’ (FCTS) control of rewards and punishments promotes cooperation best [47]. According to FCTS, the incentive institution should provide a reward if the cooperation level is lower than 0.5 and provide punishment if the cooperation level is higher than 0.5. Our study confirms that this adaptive institution also works when players make mistakes, because reward promotes cooperation best in a selfish population and punishment performs best in an altruistic population (see figure 2*a*,*b*). However, FCTS does not always maximize the group average fitness. For intermediate and large *μ*, IR is the most efficient incentive even though IP promotes cooperation better than IR in a cooperative population.

In summary, our research deepens the understanding into the influences of reward and punishment in curbing selfishness and improving social welfare. Previous research indicated that institutional punishment is a cheaper and more reliable way of inducing cooperation than is institutional reward [44,46]. However, when individuals are boundedly rational and may make mistakes, we show that institutional reward is not only effective but also efficient. This finding demonstrates the competitive advantage of reward institution in the evolutionary process and highlights the role of positive incentives in promoting cooperation. Our results also suggest that in mechanism design, uncertainty should be taken into account because the optimal incentive could be sensitive to decision errors.

## Supplementary Material

Supporting information for “The competitive advantage of institutional reward”

## Supplementary Material

Matlab code flie

## Data accessibility

The simulation code supporting this study has been uploaded as part of the electronic supplementary material in pdf formats.
