# Supplementary material for: The competitive advantage of institutional reward
Source: Proc Biol Sci. 2019 Mar 27;286(1899):20190001. doi: 10.1098/rspb.2019.0001 (PMC6452080; doi:10.1098/rspb.2019.0001)
Supplement: Supporting information for “The competitive advantage of institutional reward” [file rspb20190001supp1.pdf]

Supporting information for "The competitive advantage of institutional reward"

Yali Dong[1], Tatsuya Sasaki[2], Boyu Zhang[3*]

[1]School of Systems Science, Beijing Normal University, Beijing, 100875, China,

[2] F-power Inc., Roppongi 1-8-7-2F, Minato, Tokyo, 106-0032, Japan,

[3] Laboratory of Mathematics and Complex Systems, Ministry of Education, School of Mathematical Sciences, Beijing Normal University, Beijing, 100875, China.

1 Standard PGG with institutional incentives

1.1 Payoff calculation

We derive the expected payoffs for cooperators and defectors in PGG with institutional incentives. Let x denotes the frequency of cooperators in a population. In each time step, a sample of size n is randomly chosen to form a PGG. Thus, the probability that a focal player has k cooperators among the $n-1$ coplayers is $C_{n-1}^{k}x^{k}(1-x)^{(n-1-k)}$, where C_{n-1}^{k} is the binomial coefficient. If the focal player is a cooperator, then his/her payoff is $\frac{rc(k+1)}{n}-c-C_I+\alpha\frac{C_I n}{k+1}$, where $\frac{rc(k+1)}{n}$ is the benefit from the PGG and $\alpha\frac{C_I n}{k+1}$ is the reward. In contrast, if the focal player is a defector, then his/her payoff is $\frac{rck}{n}-C_I-(1-\alpha)\frac{C_I n}{n-k}$, where $(1-\alpha)\frac{C_I n}{n-k}$ is the punishment. Thus, the expected payoffs for a cooperator and a defector in a randomly formed PGG with $n-1$ coplayers are

$$\begin{aligned}P_C &= \sum_{k=0}^{n-1} C_{n-1}^{k}x^{k}(1-x)^{(n-1-k)}\left(\frac{rc(k+1)}{n}-c-C_I+\alpha\frac{C_I n}{k+1}\right)\\ &= \frac{rcx(n-1)}{n}-c+\frac{cr}{n}-C_I+\alpha C_I\frac{1-(1-x)^n}{x},\end{aligned} \tag{S1}$$

---

*Author for correspondence, e-mail: zhangby@bnu.edu.cn

and

$$\begin{aligned} P_D &= \sum_{k=0}^{n-1} C_{n-1}^k x^k (1-x)^{(n-1-k)} \left(\frac{rck}{n} - C_I - (1-\alpha)\frac{C_I n}{n-k} \right) \\ &= \frac{rcx(n-1)}{n} - C_I - (1-\alpha) C_I \frac{1-x^n}{1-x}, \end{aligned} \tag{S2}$$

respectively.

1.2 Without decision errors

By substituting Eqs.(S1)-(S2) into Eq.(1), we obtain

$$\frac{dx}{dt} = \omega x(1-x)\big(-c + \frac{cr}{n} + \alpha C_I \frac{1-(1-x)^n}{x} + (1-\alpha) C_I \frac{1-x^n}{1-x}\big). \tag{S3}$$

For the case of IR, Eq.(S3) is simplified as

$$\frac{dx}{dt} = \omega x(1-x)\big(-c + \frac{cr}{n} + C_I \frac{1-(1-x)^n}{x}\big). \tag{S4}$$

It is clear that $x = 0$ is stable (i.e., $\lim\limits_{x\to 0} \frac{dx}{dt} < 0$) if and only if $C_I < \frac{c}{n} - \frac{rc}{n^2} = C_I^-$, and $x = 1$ is stable (i.e., $\lim\limits_{x\to 1} \frac{dx}{dt} > 0$) if and only if $C_I > c - \frac{rc}{n} = C_I^+$. Thus, for intermediate reward $C_I^- < C_I < C_I^+$, the two boundary equilibria are unstable. Furthermore, $\frac{1-(1-x)^n}{x} = \sum_{k=0}^{n-1}(1-x)^k$ is a decreasing function of x. This implies that if the both boundary equilibria are unstable, then Eq.(S4) has a unique globally stable interior equilibrium x_R^*, where $\frac{dx}{dt} > 0$ for $x < x_R^*$ and $\frac{dx}{dt} < 0$ for $x > x_R^*$. Since $\lim\limits_{n\to\infty} \frac{1-(1-x)^n}{x} = \frac{1}{x}$, $x_R^* = \frac{nC_I}{(n-r)c}$ for larger n and the average fitness at x_R^* is $\bar{f}_R = \omega C_I (r\frac{n-1}{n-r} - 1) + 1 - \omega$ (see Figure 1a).

For the case of IP, Eq.(S3) is simplified as

$$\frac{dx}{dt} = \omega x(1-x)\big(-c + \frac{cr}{n} + C_I \frac{1-x^n}{1-x}\big). \tag{S5}$$

Analogous to the stability analysis of IR, $x = 0$ is stable if and only if $C_I < C_I^+$, and $x = 1$ is stable if and only if $C_I > C_I^-$. Thus, for intermediate punishment $C_I^- < C_I < C_I^+$, the two boundary equilibria are bistable, where the average fitness at the defective equilibrium and the cooperative equilibrium are $\bar{f}_{P0} = -2\omega C_I + 1 - \omega$ and $\bar{f}_{P1} = \omega(c(r\frac{n-1}{n} - 1) + \frac{rc}{n} - C_I) + 1 - \omega$, respectively. Furthermore, $\frac{1-x^n}{1-x} = \sum_{k=0}^{n-1} x^k$ is a increasing function of x. This implies that Eq.(S5) has an unstable interior equilibrium

x_P^* when the both boundary equilibria are stable. Since $\lim_{n\to\infty} \frac{1-x^n}{1-x} = \frac{1}{1-x}$, x_P^* is close to $1 - \frac{nC_I}{(n-r)c}$ for larger n (see Figure 1b).

For the case of IRP, we have

$$\frac{\partial^2\left(\alpha\frac{1-(1-x)^n}{x} + (1-\alpha)\frac{1-x^n}{1-x}\right)}{\partial x^2} = \frac{\partial^2\left(\alpha\sum_{k=0}^{n-1}(1-x)^k + (1-\alpha)\sum_{k=0}^{n-1}x^k\right)}{\partial x^2}$$
$$= \alpha\sum_{k=2}^{n-1}k(k-1)(1-x)^{k-2} + (1-\alpha)\sum_{k=2}^{n-1}k(k-1)x^{k-2} > 0. \tag{S6}$$

Thus, Eq.(S3) may have at most two interior equilibria (denoted by x_{RP1}^* and x_{RP2}^*) in addition to the two boundary equilibria $x = 0$ and $x = 1$ (denoted by x_{RP0} and x_{RP1}, respectively). Specifically, the defective equilibrium $x_{RP0} = 0$ is stable if and only if $C_I < \frac{(n-r)c}{n(\alpha n+1-\alpha)}$, and the cooperative equilibrium $x_{RP1} = 1$ is stable if and only if $C_I > \frac{(n-r)c}{n(\alpha+(1-\alpha)n)}$ (see Figure 1c). Furthermore, the average fitness at $x = 0$ and $x = 1$ are $\bar{f}_{RP0} = -\omega(2-\alpha)C_I + 1 - \omega$ and $\bar{f}_{RP1} = \omega(c(r-1) - (1-\alpha)C_I) + 1 - \omega$, respectively.

We now compare the average fitness at the stable (cooperative) equilibria for IR, IP, and IRP with intermediate C_I (see Figure 1e). Firstly, $\bar{f}_{P1} > \bar{f}_R$ if and only if $C_I^- < C_I < \frac{(n-r)c(r-1)}{(n-1)r}$. This means that IP is more efficient than IR for smaller C_I. Secondly, $\bar{f}_{RP1} > \bar{f}_{P1}$ for all C_I and α, i.e., IRP is always more efficient than IP whenever the cooperative equilibrium is stable. Finally, for a given α, $\bar{f}_{RP1} > \bar{f}_R$ if and only if $C_I^- < C_I < \frac{(n-r)c(r-1)}{r(n-1)-\alpha(n-r)}$, i.e., IRP is more efficient than IR for smaller C_I.

Finally, we calculate the optimal incentive at the stable (cooperative) equilibrium for different C_I. If $C_I < C_I^-$, the defective state is the only stable equilibrium for all $\alpha \in [0,1]$. In this case, the optimal incentive is IR because $\bar{f}_{R0} = -\omega C_I + 1 - \omega > \bar{f}_{RP0} > \bar{f}_{P0}$. In contrast, if $C_I > C_I^+$, the cooperative state is the only stable equilibrium for all $\alpha \in [0,1]$. In this case, the optimal incentive is also IR because $\bar{f}_{R1} = \omega c(r-1) + 1 - \omega > \bar{f}_{RP1} > \bar{f}_{P1}$. Finally, if $C_I^- < C_I < C_I^+$, $\bar{f}_{RP1}$ reach its maximum when $\alpha_{\max} = \frac{n-(n-r)c/nC_I}{n-1}$. Thus, the optimal incentive in this case is $\mathrm{IRP}_{\max}$ (see Figure 1e).

1.3 With decision errors

By substituting Eqs.(S1)-(S2) into Eq.(2), we obtain

$$\frac{dx}{dt} = \omega x(1-x)\Bigg(-c + \frac{cr}{n} + \alpha C_I \frac{1-(1-x)^n}{x} + (1-\alpha)C_I \frac{1-x^n}{1-x}\Bigg) + \mu\Bigg(\frac{1}{2} - x\Bigg). \quad \text{(S7)}$$

When $\mu > 0$, $x = 0$ and $x = 1$ are no longer boundary equilibrium. Specifically, $\lim_{x\to 0} \frac{dx}{dt} > 0$ and $\lim_{x\to 1} \frac{dx}{dt} < 0$. This implies that Eq.(S7) has at least one stable interior equilibrium x^*. Moreover, the number of stable interior equilibria depends on both μ and ω. Notice that $\frac{\partial(dx/dt)}{\partial x} = -\mu + \omega \frac{\partial[x(1-x)(P_C - P_D)]}{\partial x}$, $\frac{\partial(dx/dt)}{\partial x}$ is decreasing in x if $\frac{\mu}{\omega} > \frac{\partial[x(1-x)(P_C-P_D)]}{\partial x}$. Thus, Eq.(S7) has a unique and globally stable interior equilibrium x^* for larger μ and smaller ω. We then study the properties of x^* for different types of incentives. Since $\frac{\partial(dx/dt)}{\partial \alpha} = \omega x(1-x)C_I(\frac{1-(1-x)^n}{x} - \frac{1-x^n}{1-x}) = \omega x(1-x)C_I \sum_{k=0}^{n-1}((1-x)^k - x^k)$, $\frac{dx}{dt}$ is increasing in α for $x < 0.5$ and decreasing in α for $x > 0.5$. This implies that if the equilibrium is unique, then the equilibrium values for the three types of incentives satisfy $0 < x^*_P < x^*_{RP} < x^*_R < 0.5$ for $C_I < \frac{cn-r}{2n(1-1/2^n)} = C^*_I$ (i.e., the condition such that $x^* < 0.5$) and $1 > x^*_P > x^*_{RP} > x^*_R > 0.5$ for $C_I > C^*_I$. Thus, IR leads to the highest cooperation level for $C_I < C^*_I$ and that IP is the most effective incentive in promoting cooperation for $C_I > C^*_I$.

2 Robustness analysis

2.1 'Others only' variant of the PGG

We first evaluate the robustness of our results with respect to an 'others only' variant of the PGG. In this variant, the contribution to the public goods does not return to the contributing player but is distributed equally among all $n-1$ other members of the group. Thus, the payoffs of a cooperator and a defector in PGG with k cooperators among the $n-1$ coplayers are $\frac{rc(k-1)}{n-1} - c - C_I + \alpha\frac{C_I n}{k+1}$ and $\frac{rck}{n-1} - (1-\alpha)\frac{C_I n}{n-k}$, respectively. The expected payoffs for a cooperator and a defector in a randomly formed PGG with $n-1$

coplayers are then written as

$$\begin{aligned}P_C &= \sum_{k=0}^{n-1} C_{n-1}^k x^k (1-x)^{(n-1-k)} (\frac{rck}{n-1} - c - C_I + \alpha \frac{C_I n}{k+1}) \\ &= rcx - c - C_I + \alpha C_I \frac{1-(1-x)^n}{x},\end{aligned} \tag{S8}$$

and

$$\begin{aligned}P_D &= \sum_{k=0}^{n-1} C_{n-1}^k x^k (1-x)^{(n-1-k)} (\frac{rck}{n-1} - C_I - (1-\alpha) \frac{C_I n}{n-k}) \\ &= rcx - C_I - (1-\alpha) C_I \frac{1-x^n}{1-x},\end{aligned} \tag{S9}$$

respectively.

For the case without decision errors, we substitute Eqs.(S8)-(S9) into Eq.(1)

$$\frac{dx}{dt} = \omega x(1-x)\Big(-c + \alpha C_I \frac{1-(1-x)^n}{x} + (1-\alpha) C_I \frac{1-x^n}{1-x} \Big). \tag{S10}$$

The dynamic properties of Eq.(S10) are similar to the case of the standard PGG Eq.(S3). That is, there exists a lower bound $C_I^- = \frac{c}{n}$ and an upper bound $C_I^+ = c$, where the defective state is globally stable for $C_I < C_I^-$ and the cooperative state is globally stable for $C_I > C_I^+$. For $C_I^- < C_I < C_I^+$; IR has an globally stable interior equilibrium, IP has two locally stable boundary equilibria, and IRP has one stable cooperative equilibrium and may have another stable interior equilibrium. We further calculate the optimal α that maximizes the group average fitness at the stable (cooperative) equilibrium. If $C_I < C_I^-$, then $x = 0$ is globally stable for all $\alpha \in [0, 1]$ and the optimal incentive is IR. If $C_I > C_I^+$, then $x = 1$ is globally stable for all $\alpha \in [0, 1]$ and the optimal incentive is also IR. Finally, if $C_I^- < C_I < C_I^+$, then the fitness of IRP reach its maximum when $\alpha = \frac{n-c/C_I}{n-1}$, i.e., the maximum α such that $x = 1$ is stable.

For the case with decision errors, we substitute Eqs.(S8)-(S9) into Eq.(2)

$$\frac{dx}{dt} = \omega x(1-x)\Big(-c + \alpha C_I \frac{1-(1-x)^n}{x} + (1-\alpha) C_I \frac{1-x^n}{1-x} \Big) + \mu\Big(\frac{1}{2} - x\Big). \tag{S11}$$

When $\mu > 0$, Eq.(S11) does not have boundary equilibria, and cooperators and defectors will coexist. Similarly as Eq.(S7), Eq.(S11) has a unique and globally stable interior equilibrium for larger μ and smaller ω. Furthermore, the equilibria of the three types of

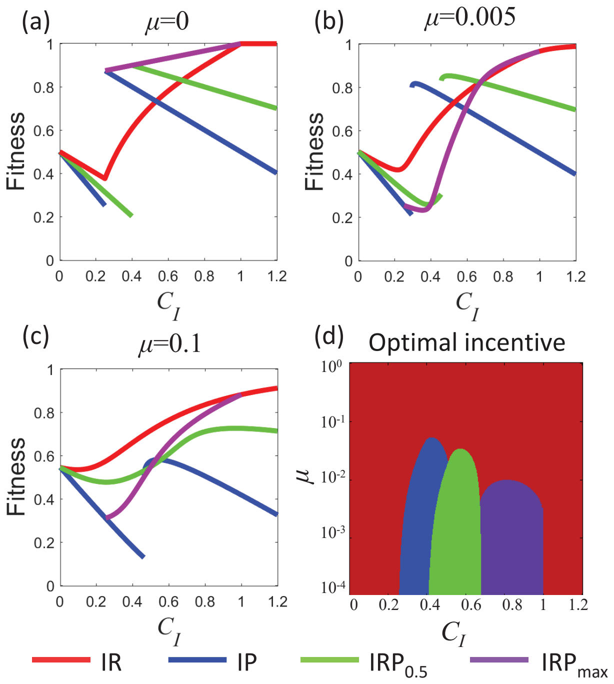

Figure S 1: 'Others only' variant of the PGG with incentives C_I and decision errors μ. The parameters are taken as $n = 4$, $c = 1$, $r = 2$, and $\omega = 0.5$. **(a)-(c)** Group average fitness at the stable (cooperative) equilibria with different types of incentives and decision errors. IRP$_{\text{max}}$ is defined for $0.25 < C_I < 1$. **(d)** Optimal incentives for different μ and C_I. IR, IP, IRP$_{0.5}$ and IRP$_{\text{max}}$ are the optimal incentives that lead to the highest group average fitness in the red, blue, green, and purple regions, respectively.

incentives satisfy $0 < x_P^* < x_{RP}^* < x_R^* < 0.5$ for $C_I < \frac{c}{2(1-1/2^n)} = C_I^*$ (i.e., the condition such that $x^* < 0.5$) and $1 > x_P^* > x_{RP}^* > x_R^* > 0.5$ for $C_I > C_I^*$. Thus, IR leads to the highest cooperation level for smaller C_I and IP is the most effective incentive in promoting cooperation for larger C_I.

The group average fitness for different types of incentives is shown in Figure S1. All the results are qualitatively the same as that of the standard PGG. Without decision errors, the optimal incentive is IR for small and large C_I and is IRP with $\alpha_{\text{max}} = \frac{n-c/C_I}{n-1}$ for intermediate C_I. With small decision errors, the optimal incentives are, from small to large C_I, IR, IP, IRP$_{0.5}$, IRP$_{\text{max}}$, and IR. With intermediate or large decision errors, the

optimal incentive is IR for all C_I.

2.2 Different types of incentive institutions

The incentive institution can be modeled in different ways. For instance, one may assume that the total amount of reward $\alpha C_I n$ is multiplied by a factor r_1 before it is distributed among cooperators, and the total amount of punishment $(1-\alpha)C_I n$ is multiplied by a factor r_2 before it is distributed among defectors. Thus, the payoff of each cooperator (or defector) is increased by $r_1\alpha\frac{C_I n}{n_C}$ (or decreased by $r_2(1-\alpha)\frac{C_I n}{n_D}$). This change does not affect our results, because we can normalize α and C_I as $\hat{\alpha} = \frac{\alpha r_1}{\alpha r_1 + (1-\alpha) r_2}$ and $\hat{C}_I = C_I(\alpha r_1 + (1-\alpha) r_2)$. In fact, the results remain unchanged as long as the expected reward for each cooperator (or punishment for each defector) decreases with the number of cooperators (defectors). Thus, our results are also robust to probabilistic incentive where only one cooperator (or defector) is exemplarily rewarded (punished).

2.3 Different types of errors

Replicator-mutator equation assumes that individuals make mistakes in strategy updating. In reality, institutions may also make mistake in incentive distribution. For instance, the institution may punish a cooperator or reward a defect due to lack of information or observation errors. To investigate how these imperfectly applied incentives might affect cooperation, we assume that the institution correctly rewards and punishes the individuals with probability $1-\varepsilon$, and randomly distribute incentives with probability ε. Thus, the expected incentive for a cooperator (or a defector) is $(1-\varepsilon)\alpha\frac{C_I n}{n_C} + \varepsilon(2\alpha - 1)C_I$ (or $(1-\varepsilon)(1-\alpha)\frac{C_I n}{n_D} + \varepsilon(2\alpha-1)C_I$), where the second term is the expected payoff that the institution randomly rewards or punishes the individual. Although the errors decrease the expected payoff of cooperators and increase the expected payoff of defectors, they do not qualitatively affects our findings because we can simply normalize C_I as $\hat{C}_I = (1-\varepsilon)C_I$. The results remain unchanged as long as the expected reward for a cooperator is more than a defector and the expected punishment for a defector is more than a cooperator.

3 Matlab codes for numerical simulations

The following Matlab code is for calculating the interior equilibria of Eq.(1). The code includes three loops. In loop 1, one can set C_I by inputting s, where $C_I = 0.001 \times (s-1)$. In particular, $s = 1 : 1001$ in Figures 1-3. In loop 2, one can set μ by inputting i, where $\mu = 0.001 \times (i-1)$. In particular, $i = 0$ in Figure 1, $i = 11$ in Figure 2(a)(c), $i = 101$ in Figure 2(b)(d), and $i = 1 : 1001$ in Figure 3. In loop 3, one can set α by inputting j, where $\alpha = 0.001 \times (j-1)$. In particular, $j = 1001$ for IR, $j = 1$ for IP, and $j = 501$ for $\text{IRP}_{0.5}$. It is worth noting that $\alpha_{\max}$ depends on C_I. Thus, for the case of $\text{IRP}_{\max}$, one should replace $\alpha(j)$ by $\frac{n-(n-r)c/(nC_I(i))}{n-1}$.

The interior equilibria of Eq.(1) are shown in 'NE'. Rows 1-3 display the largest, the second largest, and the third largest interior equilibria (for different C_I), respectively. When $\mu = 0$, the equation may have no interior equilibrium. In this case, the first row of 'NE' will display 1. When $\mu > 0$, interior equilibrium always exists and the largest one must be stable. The group average fitness at this stable equilibrium is shown in 'frp'.

```
% Matlab code starts from here
% The current parameters are for the green curve in Figure 2(b)
c=1; % set initial endowment
n=4; % set group size
w=0.5; % set selection intensity ω
r=2; % set multiplied factor r
Ci=0:0.001:1; % initialize incentive size C_I, from 0 to 1 with interval 0.001
alpha=0:0.001:1; % initialize ratio of reward α, from 0 to 1 with interval 0.001
u=0:0.001:1; % initialize decision error μ, from 0 to 1 with interval 0.001
NE=zeros(3,1001); % initialize equilibria of the replicator-mutator equation
for s=1:1001 % Loop 1: set C_I, where C_I = 0.001 × (s − 1)
    for i=101 % Loop 2: set μ, where μ = 0.001 × (i − 1)
        for j=501 % Loop 3: set α, where α = 0.001 × (j − 1)
            x=0.9999:-0.0001:0.0001; % set x
```

```
            fC=w*(r*c*x*(n-1)/n-c+c*r/n-Ci(s)+alpha(j)*Ci(s)*(1-(1-x).^n)./x)+1-w; % f_C
            fD=w*(r*c*x*(n-1)/n-Ci(s)-(1-alpha(j))*Ci(s)*(1-x.^n)./(1-x))+1-w; % f_D
            F=x.*(1-x).*(fC-fD)+u(i)/2*(1-x)-u(i)/2*x; % dx/dt
            if F(1:9998).*F(2:9999)>0 % if dx/dt > 0 or < 0 for all x, the first row displays 1
                NE(1,s)=1;
            else
                X=1-find(F(1:9998).*F(2:9999)<=0)/10000; % find the interior equilibria
                for k=1:size(X,2)
                    NE(k,s)=X(k);
                end
            end
        end
    end
end
fCC=w*(r*c*NE(1,1:1001)*(n-1)/n-c+c*r/n-Ci(1:1001)+alpha(j)*Ci(1:1001).*(1-(1-NE(1,1:1001)).^n)./NE(1,1:1001))+1-w; % fitness for cooperators at the stable (cooperative) equilibrium
fDD=w*(r*c*NE(1,1:1001)*(n-1)/n-Ci(1:1001)-(1-alpha(j))*Ci(1:1001).*(1-NE(1,1:1001).^n)./(1-NE(1,1:1001)))+1-w; % fitness for defectors at the stable (cooperative) equilibrium
frp=fCC.*NE(1,1:1001)+fDD.*(1-NE(1,1:1001)); % group average fitness at the stable (cooperative) equilibrium
plot(0:0.001:1,NE(1,1:1001),'LineWidth',2,'Color','g'); axis([0 1 0 1]); % plot an equilibrium
```
